# SARS-CoV-2 membrane glycoprotein M antagonizes the MAVS-mediated innate antiviral response

**DOI:** 10.1038/s41423-020-00571-x

**Published:** 2020-10-27

**Authors:** Yu-Zhi Fu, Su-Yun Wang, Zhou-Qin Zheng, Yi Huang, Wei-Wei Li, Zhi-Sheng Xu, Yan-Yi Wang

**Affiliations:** 1 Key Laboratory of Special Pathogens and Biosafety, Center for Biosafety Mega-Science, Wuhan Institute of Virology, Chinese Academy of Sciences, 430071 Wuhan, China; 2 University of Chinese Academy of Sciences, 100049 Beijing, China

**Keywords:** SARS-CoV-2, Membrane glycoprotein M, MAVS, Type I interferon, Innate immunity, Immune evasion, Innate immunity

## Abstract

A novel SARS-related coronavirus (SARS-CoV-2) has recently emerged as a serious pathogen that causes high morbidity and substantial mortality. However, the mechanisms by which SARS-CoV-2 evades host immunity remain poorly understood. Here, we identified SARS-CoV-2 membrane glycoprotein M as a negative regulator of the innate immune response. We found that the M protein interacted with the central adaptor protein MAVS in the innate immune response pathways. This interaction impaired MAVS aggregation and its recruitment of downstream TRAF3, TBK1, and IRF3, leading to attenuation of the innate antiviral response. Our findings reveal a mechanism by which SARS-CoV-2 evades the innate immune response and suggest that the M protein of SARS-CoV-2 is a potential target for the development of SARS-CoV-2 interventions.

## Introduction

The innate immune response is the first line of host defense against viral infection and is initiated by the recognition of pathogen-associated molecular patterns by cellular pattern recognition receptors (PRRs). This triggers a series of signaling events that lead to the induction of type I interferons (IFNs), proinflammatory cytokines, and other antiviral effector genes.^1–4^ These downstream effector proteins mediate the innate antiviral response and facilitate adaptive immunity to suppress viral replication and eliminate viruses in vivo.^5,6^

Among the PRRs, retinoic acid-inducible gene-I (RIG-I)-like receptors (RLRs), including RIG-I and melanoma differentiation associated gene 5 (MDA5), have been demonstrated to be cytosolic viral RNA sensors in mammals.^6–8^ Although RIG-I and MDA5 have a certain specificity for sensing distinct types of viral RNAs, they utilize a common adaptor protein called MAVS (also known as VISA, IPS-1 or Cardif) to trigger downstream signal transduction.^9–12^ After binding viral RNA, RLRs are recruited to the mitochondria-located MAVS via their respective CARD domains,^1^ leading to the aggregation and formation of prion-like MAVS polymers.^13^ The aggregated MAVS acts as a central platform for the recruitment of downstream signaling components, including TRAF2/3/5/6, TBK1 and IKK, leading to the activation of the transcription factors IRF3 and NF-κB and to the induction of downstream antiviral effector genes.^14–17^

SARS-CoV-2, a member of the coronavirus family, is a typical single-stranded positive-sense RNA virus that encodes over 28 proteins, including 4 structural proteins (spike, membrane, envelope, and nucleocapsid), 16 nonstructural proteins (NSP1-NSP16), and 8 auxiliary proteins (ORF3a, ORF3b, ORF6, ORF7a, ORF7b, ORF8, ORF9b and ORF14).^18–20^ Recently, SARS-CoV-2 has caused a global pandemic and worldwide social and economic disruption. Patients with severe SARS-CoV-2 infection usually have dyspnea after 1 week, which then rapidly manifests as acute respiratory distress syndrome and results in death.^21–25^ Dysregulation of IFNs has been found in patients with severe COVID-19.^26,27^ However, little is known about how SARS-CoV-2 evades the immune system.

SARS-CoV-2 membrane protein M is a 222-amino acid glycosylated structural protein with three N-terminal membrane-spanning domains, which are essential for the assembly of viral particles.^18^ In this study, we identified the SARS-CoV-2 M protein as an inhibitor of the MAVS-mediated innate antiviral immune response. Our results suggest that M targets MAVS and impairs its aggregation and recruitment of downstream signaling components. Our findings reveal a mechanism by which SARS-CoV-2 evades the innate immune response.

## Results

### SARS-CoV-2 protein M antagonizes the viral RNA-triggered innate antiviral immune response

**Fig. 1:**
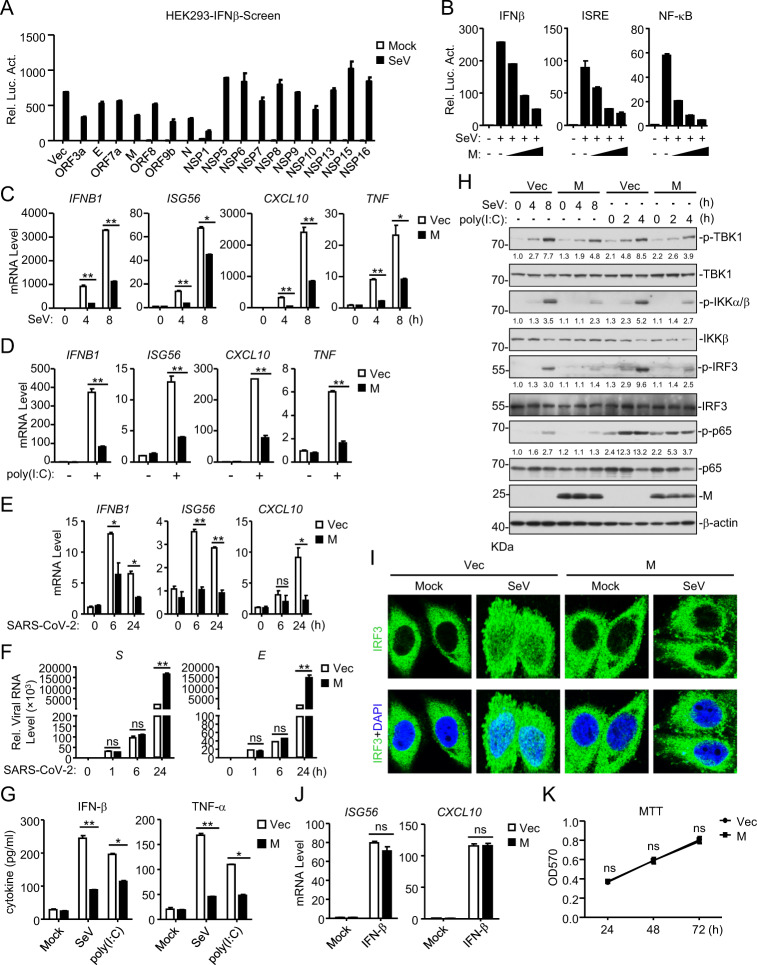
Identification of SARS-CoV-2 M as an inhibitor of the viral RNA-triggered innate immune response. **a** Screening for SARS-CoV-2 proteins that inhibit the SeV-triggered activation of the IFN-β promoter. HEK293T cells were transfected with an IFN-β promoter luciferase plasmid and the indicated SARS-CoV-2 protein expression plasmids for 20 h and then infected with SeV (MOI = 1) or left untreated for 12 h before luciferase assays were performed. **b** The M protein inhibits the SeV-triggered activation of the IFNβ promoter, ISRE and NF-κB in a dose-dependent manner. HEK293T cells were transfected with the indicated reporter plasmids and increasing amounts of the Flag-M plasmid for 20 h and then infected with SeV (MOI = 1) or left untreated for 12 h before luciferase assays were performed. **c** The M protein inhibits the SeV-triggered transcription of antiviral genes in HEK293 cells. HEK293 cells stably expressing the M protein were left uninfected or infected with SeV (MOI = 1) for the indicated times before qPCR analysis was performed. **d** The M protein inhibits the poly (I:C)-triggered transcription of antiviral genes in HEK293 cells. HEK293 cells stably expressing the M protein were mock-transfected or transfected with poly (I:C) for 6 h before qPCR analysis was performed. **e** The M protein inhibits the SARS-CoV-2-triggered transcription of antiviral genes in HEK293 cells. HEK293-ACE2 cells were transfected with the Flag-M plasmid for 20 h and then infected with SARS-CoV-2 (MOI = 1) or left uninfected for the indicated times before qPCR analysis was performed. **f** Effects of M on viral genome replication during SARS-CoV-2 infection in HEK293-ACE2 cells. HEK293-ACE2 cells were transfected with the Flag-M plasmid for 20 h and then infected with SARS-CoV-2 (MOI = 1) or left uninfected for the indicated times before qPCR analysis was performed. **g** The M protein inhibits the SeV- and poly (I:C)-induced secretion of IFN-β and TNF-α in HEK293 cells. HEK293 cells stably expressing the M protein were infected with SeV (MOI = 1) or transfected with poly (I:C) for 8 h before measurement of IFN-β and TNF-α by ELISA. **h** The M protein impairs the SeV- and poly (I:C)-induced phosphorylation of downstream components. HEK293 cells stably expressing the M protein were infected with SeV (MOI = 1) or transfected with poly (I:C) for the indicated times before immunoblot analysis was performed. **i** M impairs the SeV-induced nuclear translocation of IRF3. HeLa cells were transfected with the Flag-M plasmid for 20 h and then infected with SeV (MOI = 1) or left uninfected for 10 h before confocal microscopy was performed. **j** Effects of the M protein on the IFN-β-induced transcription of the *ISG56* and *CXCL10* genes. HEK293 cells stably expressing the M protein were untreated or treated with IFN-β for 4 h before qPCR analysis was performed. **k** Effects of the M protein on cell viability. HEK293 cells stably expressing the M protein and vector were measured by MTT assays. Graphs show the mean ± SD; *n* = 3; ns not significant; *p < 0.05, **p < 0.01 (Student’s unpaired *t* test)

It has been demonstrated that RLRs mediate the innate immune response to RNA viruses, including SARS-CoV.^28–30^ To identify SARS-CoV-2 proteins that may inhibit the RLR-mediated induction of downstream antiviral genes, we constructed 17 SARS-CoV-2 protein expression clones and screened for candidates that inhibit the Sendai virus (SeV, an RNA virus)-induced activation of the IFNβ promoter in HEK293 cells by reporter assays (Fig. 1A). This screen identified the SARS-CoV-2 ORF3a, M, N, ORF9b and NSP1 proteins as candidates. In reporter assays, ectopic expression of the M protein dose-dependently inhibited the SeV-induced activation of the IFNβ promoter, ISRE and NF-κB in HEK293T cells (Fig. 1B). To further confirm the role of M, we established stable HEK293 cells ectopically expressing M (HEK293-M) by lentiviral transduction. qPCR analysis indicated that ectopic expression of M inhibited the transcription of the *IFNB1*, *ISG56*, *CXCL10* and *TNF* genes induced by SeV infection (Fig. 1C) or transfection of the synthetic RNA analog poly (I:C) (Fig. 1D) in HEK293-M cells. Similarly, overexpression of M markedly suppressed the SARS-CoV-2-induced transcription of *IFNB1* and downstream antiviral genes at 6 and 24 h post infection in HEK293 cells stably expressing ACE2 (HEK293-ACE2) (Fig. 1E). To further examine whether the inhibitory effect of M on type I IFN induction is due to an indirect influence on viral replication, we assessed the role of M in SARS-CoV-2 replication by qPCR. As shown in Fig. 1F, M barely affected SARS-CoV-2 replication at 1 and 6 h post infection, indicating that M inhibited the transcription of antiviral genes without affecting viral replication. Twenty-four hours post infection, enhanced viral genome replication was observed in cells overexpressing M. This enhancement was probably caused by the M-mediated inhibition of the cellular antiviral response since the RLR-mediated antiviral innate immune response is well known to be essential for the inhibition of viral replication at the early stage of infection.^2,10–12^ We next performed ELISA experiments and found that the secretion of IFN-β and TNF-α following SeV infection or poly (I:C) transfection was also impaired in HEK293-M cells (Fig. 1G). In addition, M inhibited the phosphorylation of TBK1, IKKα/β, IRF3 and p65 induced by SeV or poly(I:C) (Fig. 1H). Consistently, confocal microscopy showed that compared with that in the control cells, the SeV-triggered nuclear translocation of IRF3 was disrupted in cells ectopically expressing M (Fig. 1I). In contrast, M had no marked effects on the IFN-β-induced transcription of the *ISG56* and *CXCL10* genes (Fig. 1J). Since the above experiments were performed with 293 cells stably expressing M, we further evaluated whether M affected cell viability. Cell proliferation assays confirmed that M had no effects on cell viability (Fig. 1K). Taken together, these results suggest that M acts as an inhibitor of the RNA virus-triggered innate immune response.

### M inhibits the MAVS-mediated induction of downstream antiviral genes

**Fig. 2:**
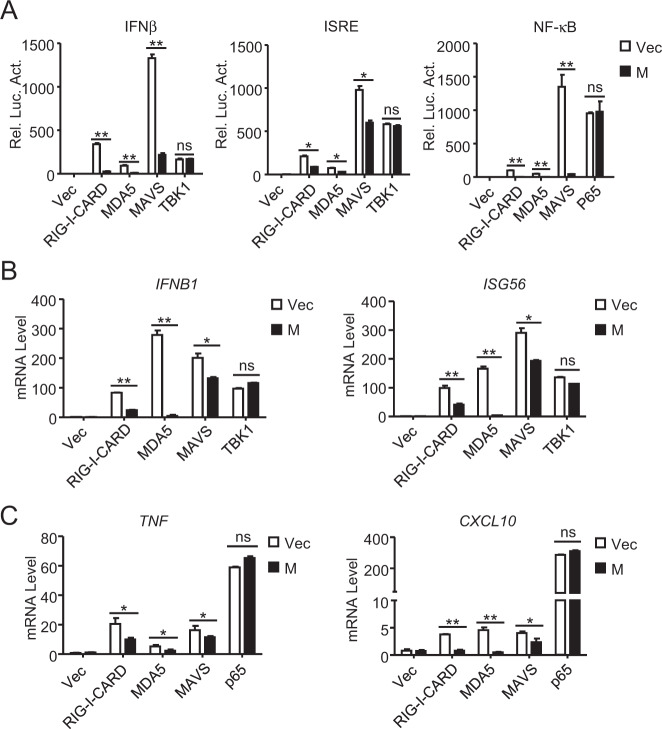
The M protein negatively regulates MAVS-mediated signaling. **a** Effects of the M protein on activation of the IFNβ promoter, ISRE and NF-κB mediated by various components. HEK293 cells were transfected with the IFNβ promoter, ISRE or NF-κB reporter, M plasmid and the indicated expression plasmids for 20 h before luciferase assays were performed. **b**, **c** Effects of the M protein on antiviral gene transcription mediated by various components. HEK293 cells were transfected with M and the indicated expression plasmids for 15 h before qPCR analysis was performed. Graphs show the mean ± SD, *n* = 3. ns not significant, *p < 0.05, **p < 0.01 (Student’s unpaired *t* test)

We next investigated the mechanisms underlying the M-mediated inhibition of the innate antiviral response. Reporter assays indicated that M inhibited the activation of the IFNβ promoter and ISRE mediated by overexpression of RIG-I-CARD, MDA5 and MAVS but not their downstream component TBK1 (Fig. 2A). M also inhibited the NF-κB activation mediated by RIG-I-CARD, MDA5 and MAVS but not downstream p65 in HEK293 cells (Fig. 2A). Consistently, qPCR analysis showed that ectopic expression of M inhibited the IRF3-controlled transcription of the *IFNB1* and *ISG56* genes induced by overexpression of RIG-I-CARD, MDA5 and MAVS but not TBK1 (Fig. 2B). In similar experiments, M also inhibited the transcription of the NF-κB-controlled *TNF* and *CXCL10* genes induced by overexpression of RIG-I-CARD, MDA5 and MAVS but not p65 (Fig. 2C). These results suggest that M inhibits innate antiviral signaling at the MAVS level.

**Fig. 3:**
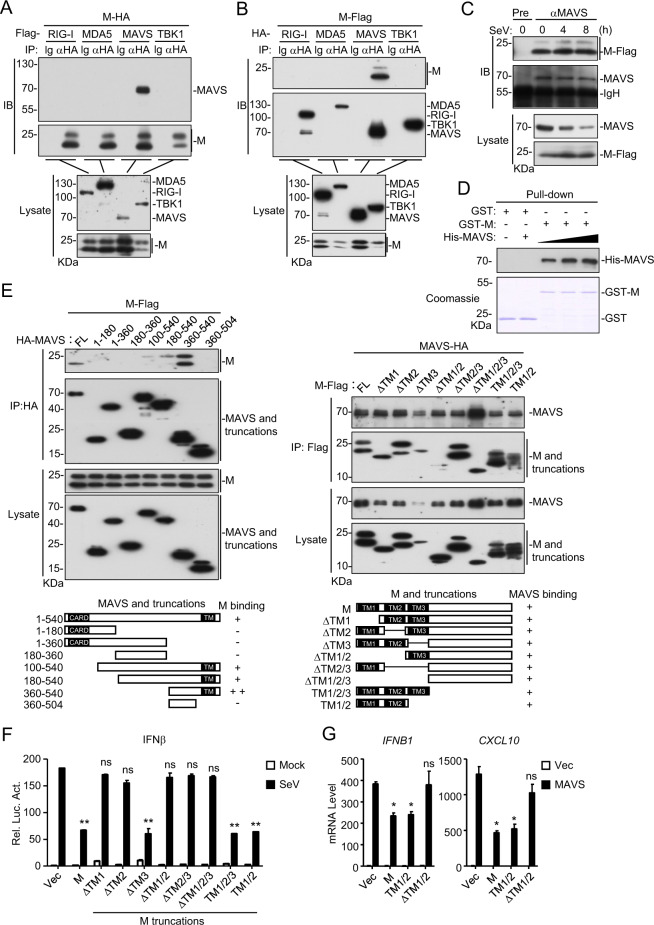
Interaction of M with MAVS. **a**, **b** The M protein interacts with MAVS in the mammalian overexpression system. HEK293T cells were transfected with the indicated plasmids for 20 h. Coimmunoprecipitation and immunoblot analyses were performed with the indicated antibodies. **c** Association of the M protein with endogenous MAVS. Flag-tagged M-expressing HEK293 cells were infected with SeV (MOI = 1) for the indicated times. Coimmunoprecipitation and immunoblot analyses were performed with the indicated antibodies. **d** The M protein directly binds to MAVS. Purified recombinant GST-M was bound to glutathione agarose beads and incubated with recombinant His-MAVS for 3 h. The bead-bound proteins were analyzed by immunoblotting with the indicated antibodies. **e** Domain mapping of the M-MAVS association. HEK293 cells were transfected with the indicated M and MAVS truncation mutants for 20 h before coimmunoprecipitation and immunoblotting analyses were performed with the indicated antibodies. **f** Effects of M truncations on the SeV-induced activation of the IFNβ promoter. HEK293 cells were transfected with the IFNβ promoter luciferase plasmid and the indicated expression plasmids for 20 h. The cells were then infected with SeV (MOI = 1) or left uninfected for 12 h before luciferase assays were performed. **g** Effects of M truncations on the MAVS-mediated transcription of downstream antiviral genes. HEK293 cells were transfected with the indicated plasmids for 15 h before qPCR analysis was performed. Graphs show the mean ± SD; *n* = 3; ns not significant; *p < 0.05, **p < 0.01 (Student’s unpaired *t* test)

Coimmunoprecipitation experiments indicated that M specifically associated with MAVS but not RIG-I, MDA5 or TBK1 in the mammalian overexpression system (Fig. 3A, B). Further experiments indicated that the M protein was constitutively associated with endogenous MAVS before and after SeV infection in HEK293 cells (Fig. 3C). In vitro GST pull-down assays with recombinant proteins indicated that the M protein directly interacted with MAVS in a dose-dependent manner (Fig. 3D). Domain-mapping experiments indicated that the transmembrane domain of MAVS was required for its association with M. Among the examined truncations, MAVS (360–540) interacted with the M protein most strongly, while MAVS (180–540) and MAVS (100–540) interacted with the M protein weakly, suggesting that the middle fragment (aa100–360) of MAVS inhibits its interaction with the M protein (Fig. 3E). All the examined M truncations, including TM1/2 (contained only the two N-terminal transmembrane domains) and ΔTM1/2 (lacked the two N-terminal transmembrane domains), interacted with MAVS (Fig. 3E), suggesting that the M protein could interact with MAVS via its different fragments. Reporter assays indicated that the two N-terminal transmembrane domains of the M protein were required for its inhibition of SeV-induced IFNβ promoter activation (Fig. 3F) as well as for the MAVS-mediated transcription of the downstream *IFNB1* and *CXCL10* genes (Fig. 3G). A possible explanation is that although multiple motifs of M could mediate its association with VISA, only the TM1/2 domains inhibit the activation of VISA. Taken together, these results suggest that M inhibits the innate antiviral response by targeting MAVS, and its TM1/2 domains are essential for this inhibitory function.

### M impairs MAVS aggregation and its recruitment of downstream components

**Fig. 4:**
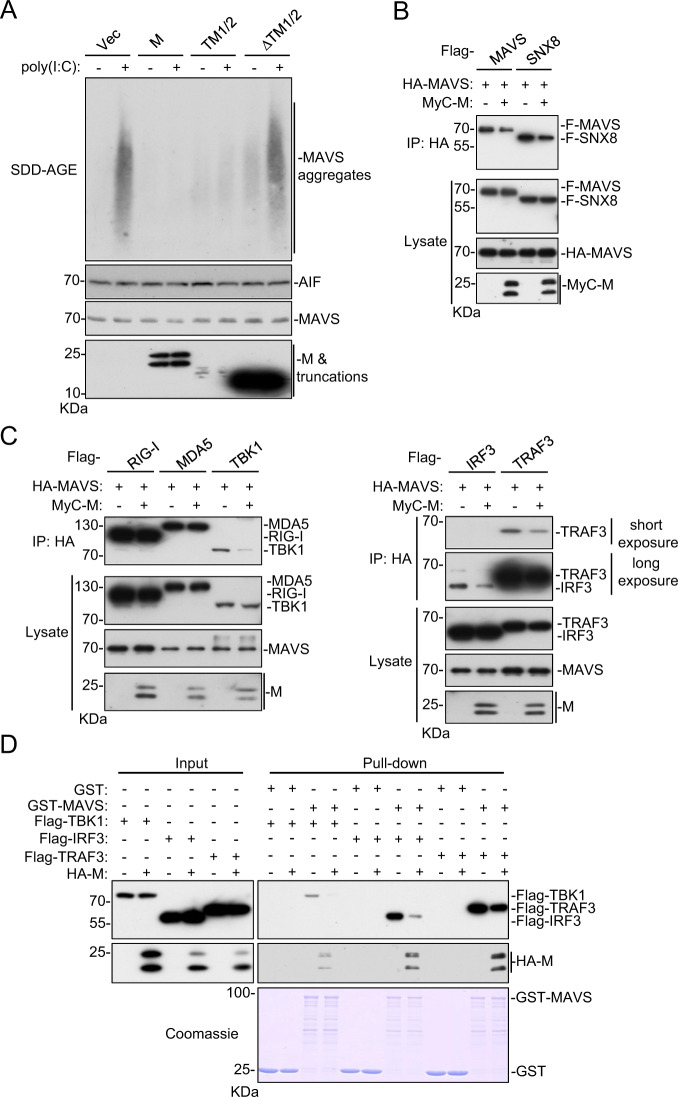
The M protein inhibits MAVS aggregation and its recruitment of downstream components. **a** Overexpression of M and TM1/2 inhibits the poly(I:C)-triggered aggregation of MAVS. Crude mitochondrial extracts were prepared from HEK293 cells transfected with the indicated plasmids for 20 h and then transfected with poly (I:C) for 8 h. The extracts and whole-cell lysates were fractionated by SDD-AGE and SDS-PAGE before immunoblot analysis with the indicated antibodies. **b** Effects of the M protein on MAVS-SNX8 interaction⊡ HEK293T cells were transfected with the indicated plasmids for 20 h before coimmunoprecipitation and immunoblot analyses were performed with the indicated antibodies. **c** The M protein disrupts the recruitment of TRAF3, TBK1 and IRF3 to the MAVS complex. HEK293T cells were transfected with the indicated plasmids for 20 h before coimmunoprecipitation and immunoblot analyses were performed with the indicated antibodies. **d** The M protein impairs the interaction of MAVS with TRAF3, TBK1 and IRF3 in vitro. Purified recombinant GST-MAVS was bound to glutathione agarose beads and incubated with lysate of HEK293 cells transfected with Flag-TRAF3, Flag-TBK1, Flag-IRF3 and/or M-HA as indicated. The bead-bound proteins were analyzed by immunoblotting with the indicated antibodies

The prion-like aggregation of MAVS was previously reported to be essential for activating the innate antiviral response after viral infection.^13^ We investigated whether M impairs the formation of MAVS aggregates. As shown in Fig. 4A, semidenaturing detergent agarose gel electrophoresis (SDD-AGE) analysis indicated that the M protein and its TM1/2 truncation but not the ΔTM1/2 truncation impaired the aggregation of MAVS induced by poly (I:C) transfection. Previously, SNX8 was reported to be indispensable for MAVS aggregation after virus infection.^31^ As shown in Fig. 4B, the M protein disrupted the self-association of MAVS and its association with SNX8. These results suggest that the M protein impairs the innate antiviral response by inhibiting MAVS aggregation.

Upon aggregation, MAVS acts as a central platform to recruit downstream signaling components such as TRAF3, TBK1 and IRF3, leading to the subsequent induction of downstream antiviral genes.^16^ Consistently, coimmunoprecipitation experiments indicated that the M protein disrupted the recruitment of TRAF3, TBK1 and IRF3 to the MAVS complex but had no marked effects on the interaction between MAVS and RIG-I or MDA5 (Fig. 4C). In vitro GST pull-down assays showed that the M protein inhibited the binding of MAVS to TRAF3, TBK1 and IRF3 (Fig. 4D). These results suggest that the M protein impairs the recruitment of TRAF3, TBK1 and IRF3 to the MAVS complex, leading to the inhibition of the innate antiviral response.

## Discussion

During coevolution with hosts, viruses have evolved multiple mechanisms to evade host immune defense.^32–34^ How SARS-CoV-2 evades the host immune defense is not well understood. In this study, we identified the SARS-CoV-2 M protein as a factor underlying the inhibition of host antiviral innate immunity by directly targeting the central adaptor MAVS in the RLR-mediated induction of type I IFNs.

Several lines of evidence suggest that M directly targets MAVS to inhibit the innate immune response. The M protein specifically inhibited the SARS-CoV-2-, SeV- and poly (I:C)-induced transcription of downstream antiviral genes. The M protein suppressed signaling mediated by RIG-I, MDA5 and MAVS but not their downstream components TBK1 or p65. Coimmunoprecipitation and in vitro pull-down assays indicated that the M protein directly interacted with MAVS. Further studies suggested that the M protein impaired MAVS aggregation induced by viral RNA as well as its recruitment of downstream components such as TRAF3, TBK1, and IRF3. These results suggest that the M protein evades the innate antiviral response by impairing MAVS activation.

Previously, SARS-CoV and MERS-CoV were shown to have developed multiple mechanisms to evade the innate immune response. For example, SARS-CoV M, N, 9b, and NSP1 as well as MERS-CoV M, ORF4a, and ORF4b have been demonstrated to target different signaling components in the innate immune response pathways.^28,29,35,36^ Some recent screenings have identified SARS-CoV-2 M, N, ORF3a, ORF6, NSP1,NSP3, NSP12, NSP13, NSP14 and NSP15 as potential candidates for inhibiting the induction of IFNβ .^37,38^ In particular, it has been shown that the SARS-CoV M protein impairs the innate immune response by inhibiting the TRAF3/TANK/TBK1 complex,^28,35^ whereas the MERS-CoV M protein impairs the TBK1-mediated phosphorylation of IRF3.^39^ Considering these results and those of our studies, it is possible that the SARS-CoV family of viruses utilizes their M proteins as a general strategy to evade the host innate immune system. Our current findings facilitate the understanding of the complicated interaction between SARS-CoV-2 and the host and provide a potential target for the development of interventions for SARS-CoV-2.

## Materials and methods

### Reagents, antibodies, virus, and cells

The following reagents were purchased from the indicated manufacturers: Lipofectamine 2000 (Invitrogen); puromycin (Thermo); Dual-specific luciferase assay kit (Promega); SYBR (BIO-RAD); polybrene (Millipore); MTT (MedChemExpress); ELISA kit for human IFN-β and TNF-α (4A Biotech); mouse antibodies against Flag and β-actin (Sigma), His and HA (OriGene), MyC (9B11), and IKKβ (8943S) (Cell Signaling Technology); rabbit monoclonal antibodies against HA, phosphor-IKKα (Ser176)/β (Ser177) (2078S) (Cell Signaling Technology), phosphor TBK1 (ab109272) and TBK1 (ab40676) (Abcam); and rabbit polyclonal antibodies against MAVS (Bethyl). HEK293, HeLa and HEK293T cells were purchased from ATCC. HEK293 cells stably expressing ACE2 (HEK293-ACE2) were obtained by lentiviral transduction. SeV (Cantell strain) was obtained as previously described.^40^ SARS-CoV-2 (IVCAS 6.7512) was isolated from BALF collected from a patient with viral pneumonia in December 2019 in Wuhan, China.^41^ Isolated viral particles were propagated in Vero E6 cells.^42^ HEK293-ACE2 cells were infected with SARS-CoV-2 in a biosafety level 4 (BSL-4) laboratory (Wuhan).

### Plasmid construction

The IFNβ promoter, ISRE and NF-κB luciferase reporter plasmids, and mammalian expression plasmids for Flag- and HA-tagged RIG-I, MDA5, MAVS, TRAF3, and TBK1 were previously described^14,43^ Flag-, MyC-, and HA-tagged M and their mutants were constructed by standard molecular biology techniques.

### Transfection and reporter assays

Luciferase assays were performed using a dual-specific luciferase assay kit (Promega). Cells (1 × 10^5^) were seeded in 48-well plates and transfected the following day by standard calcium phosphate precipitation. In the same experiment, empty control plasmid was added to ensure that the same amount of total DNA was used for each transfection. To normalize for transfection efficiency, 0.01 μg of the pRL-TK (*Renilla* luciferase) reporter plasmid was added to each transfection.^31^

### Real-time PCR

Total RNA was isolated for qPCR analysis to measure the mRNA levels of the indicated genes. The data shown are the relative abundances of the indicated mRNAs normalized to that of GAPDH. Primer sequences for *IFNB1*, *ISG56*, *CXCL10*, *TNF*, and GAPDH were previously described.^31,40^ The SARS-CoV-2 *S* primers CTTCCCTCAGTCAGCACCTC (forward) and AACCAGTGTGTGCCATTTGA (reverse) as well as the SARS-CoV-2 *E* primers TCGTTTCGGAAGAGACAGGT (forward) and CACGAGAGTAAACGTAAAAAGAAGG (reverse) were utilized.

### ELISA

HEK293-M or control cells were infected with SeV or poly (I:C) for 8 h. The culture media were collected for measurement of IFN-β and TNF-α by ELISA.

### Coimmunoprecipitation and immunoblot analysis

Coimmunoprecipitation and immunoblot analyses were performed as previously described.^34,44^ In brief, for coimmunoprecipitation, cells (1 × 10^7^) were lysed in 1 mL of NP-40 lysis buffer (20 mM Tris-HCl [pH 7.4], 150 mM NaCl, 1 mM EDTA, 1% Nonidet P-40, 10 μg/mL aprotinin, 10 μg/mL leupeptin, and 1 mM phenylmethylsulfonyl fluoride). For direct analysis of protein expression, cells were lysed with SDS-PAGE loading buffer and then ultrasonicated.

### GST pull-down assay

GST-M was bound to glutathione agarose beads and incubated with purified His-MAVS or lysate of HEK293T cells transiently expressing Flag-TBK1, Flag-IRF3, or Flag-TRAF3 along with an empty or HA-M plasmid for 3 h. The beads were collected and washed three times with lysis buffer (20 mM Tris-HCl [pH 7.4], 150 mM NaCl, 1 mM EDTA, 1% NP-40, 10 μg/mL aprotinin, 10 μg/mL leupeptin, 1 mM phenylmethylsulfonyl fluoride). The beads were then mixed with SDS loading buffer and boiled for 10 min. The input/eluates were resolved by SDS-PAGE and analyzed by Coomassie staining and/or immunoblot.

### Semidenaturing detergent agarose gel electrophoresis (SDD-AGE)

Semidenaturing detergent agarose gel electrophoresis (SDD-AGE) was performed as previously described.^14^ In brief, crude mitochondria were resuspended in 1× sample buffer (0.5× TBE, 10% glycerol, 2% SDS, and 0.0025% bromophenol blue) and loaded onto a vertical 1.5% agarose gel (Bio-Rad). After electrophoresis in running buffer (1×TBE and 0.01% SDS) for 50 min with a constant voltage of 100 V at 4 °C, the proteins were transferred to an immobile membrane (Millipore) for immunoblot analysis.

### MTT assay

The cells were plated in 96-well culture plates (2000 cells/200 µL/well for all cell lines) and incubated in a 5% CO2 incubator. Further, at the respective time points, 10 µL of the stock MTT solution (5 mg/mL) was added, and the cells were incubated in a 5% CO2 incubator for 4 h. The medium was removed, and formalin crystals formed by the cells were dissolved in 100 µL of DMSO. The absorbance was read at a 570 nm wavelength on a multiwell plate reader.

### Statistics

Unpaired Student’s *t* test was used for statistical analysis with GraphPad Prism software; *P < 0.05 and **P < 0.01 were considered significant, and ns indicates no significance.

## References

[1] Hu MM, Shu HB (2018). Cytoplasmic mechanisms of recognition and defense of microbial nucleic acids. Annu. Rev. Cell Dev. Biol..

[2] Wu J, Chen ZJ (2014). Innate immune sensing and signaling of cytosolic nucleic acids. Annu. Rev. Immunol..

[3] Akira S, Uematsu S, Takeuchi O (2006). Pathogen recognition and innate immunity. Cell.

[4] Takeuchi O, Akira S (2010). Pattern recognition receptors and inflammation. Cell.

[5] Barbalat R, Ewald SE, Mouchess ML, Barton GM (2011). Nucleic acid recognition by the innate immune system. Annu. Rev. Immunol..

[6] Kang DC (2002). mda-5: An interferon-inducible putative RNA helicase with double-stranded RNA-dependent ATPase activity and melanoma growth-suppressive properties. Proc. Natl Acad. Sci. USA.

[7] Yoneyama M (2004). The RNA helicase RIG-I has an essential function in double-stranded RNA-induced innate antiviral responses. Nat. Immunol..

[8] Andrejeva J (2004). The V proteins of paramyxoviruses bind the IFN-inducible RNA helicase, mda-5, and inhibit its activation of the IFN-beta promoter. Proc. Natl Acad. Sci. USA.

[9] Kawai T (2005). IPS-1, an adaptor triggering RIG-I- and Mda5-mediated type I interferon induction. Nat. Immunol..

[10] Meylan E (2005). Cardif is an adaptor protein in the RIG-I antiviral pathway and is targeted by hepatitis C virus. Nature.

[11] Seth RB, Sun L, Ea CK, Chen ZJ (2005). Identification and characterization of MAVS, a mitochondrial antiviral signaling protein that activates NF-kappaB and IRF 3. Cell.

[12] Xu LG (2005). VISA is an adapter protein required for virus-triggered IFN-beta signaling. Mol. Cell.

[13] Hou F (2011). MAVS forms functional prion-like aggregates to activate and propagate antiviral innate immune response. Cell.

[14] Yan BR (2017). PKACs attenuate innate antiviral response by phosphorylating VISA and priming it for MARCH5-mediated degradation. Plos Pathog..

[15] Yoneyama M, Fujita T (2009). RNA recognition and signal transduction by RIG-I-like receptors. Immunol. Rev..

[16] Mao AP (2010). Virus-triggered ubiquitination of TRAF3/6 by cIAP1/2 is essential for induction of interferon-beta (IFN-beta) and cellular antiviral response. J. Biol. Chem..

[17] Wang YY (2010). WDR5 is essential for assembly of the VISA-associated signaling complex and virus-triggered IRF3 and NF-kappaB activation. Proc. Natl Acad. Sci. USA.

[18] Gordon, D. E. et al. A SARS-CoV-2-human protein-protein interaction map reveals drug targets and potential drug-repurposing. bioRxiv: the preprint server for biology (2020). https://www.biorxiv.org/content/10.1101/2020.03.22.002386v3.

[19] Blanco-Melo D (2020). Imbalanced Host Response to SARS-CoV-2 Drives Development of COVID-19. Cell.

[20] Wu A (2020). Genome Composition and Divergence of the Novel Coronavirus (2019-nCoV) Originating in China. Cell Host Microbe.

[21] Tay MZ, Poh CM, Renia L, MacAry PA, Ng LFP (2020). The trinity of COVID-19: immunity, inflammation and intervention. Nat. Rev. Immunol..

[22] Huang C (2020). Clinical features of patients infected with 2019 novel coronavirus in Wuhan, China. Lancet.

[23] Wang Z, Yang B, Li Q, Wen L, Zhang R (2020). Clinical Features of 69 Cases with Coronavirus Disease 2019 in Wuhan, China. Clin. Infect. Dis..

[24] Wu F (2020). A new coronavirus associated with human respiratory disease in China. Nature.

[25] Gudbjartsson DF (2020). Spread of SARS-CoV-2 in the Icelandic Population. N. Engl. J. Med..

[26] Trouillet-Assant S (2020). Type I IFN immunoprofiling in COVID-19 patients. J. Allergy Clin. Immunol..

[27] Hadjadj J (2020). Impaired type I interferon activity and inflammatory responses in severe COVID-19 patients. Science.

[28] Siu KL (2009). Severe acute respiratory syndrome coronavirus M protein inhibits type I interferon production by impeding the formation of TRAF3.TANK.TBK1/IKKepsilon complex. J. Biol. Chem..

[29] Park A, Iwasaki A (2020). Type I. and Type III Interferons - Induction, Signaling, Evasion, and Application to Combat COVID-19. Cell Host Microbe..

[30] Subbarao K, Mahanty S (2020). Respiratory Virus Infections: Understanding COVID-19. Immunity.

[31] Guo, W. et al. SNX8 modulates the innate immune response to RNA viruses by regulating the aggregation of VISA. *Cell Mol. Immunol.*10.1038/s41423-019-0285-2 (2019). 10.1038/s41423-019-0285-2 PMC7784681 31511639

[32] Hu MM, Shu HB (2020). Innate immune response to cytoplasmic DNA: mechanisms and diseases. Annu. Rev. Immunol..

[33] Fu YZ (2019). Human cytomegalovirus protein UL42 antagonizes cGAS/MITA-mediated innate antiviral response. Plos Pathog..

[34] Fu YZ (2019). Human Cytomegalovirus DNA Polymerase Subunit UL44 Antagonizes Antiviral Immune Responses by Suppressing IRF3- and NF-kappaB-Mediated Transcription. J. Virol..

[35] Siu KL, Chan CP, Kok KH, Chiu-Yat Woo P, Jin DY (2014). Suppression of innate antiviral response by severe acute respiratory syndrome coronavirus M protein is mediated through the first transmembrane domain. Cell. Mol. Immunol..

[36] Kamitani W (2006). Severe acute respiratory syndrome coronavirus nsp1 protein suppresses host gene expression by promoting host mRNA degradation. Proc. Natl Acad. Sci. USA.

[37] Lei X (2020). Activation and evasion of type I interferon responses by SARS-CoV-2. Nat. Commun..

[38] Yuen CK (2020). SARS-CoV-2 nsp13, nsp14, nsp15 and orf6 function as potent interferon antagonists. Emerg. Microbes Infect..

[39] Lui PY (2016). Middle East respiratory syndrome coronavirus M protein suppresses type I interferon expression through the inhibition of TBK1-dependent phosphorylation of IRF3. Emerg. Microbes Infect..

[40] Zou HM (2020). Human Cytomegalovirus Protein UL94 Targets MITA to Evade the Antiviral Immune Response. J. Virol..

[41] Zhou P (2020). A pneumonia outbreak associated with a new coronavirus of probable bat origin. Nature.

[42] Jiang RD (2020). Pathogenesis of SARS-CoV-2 in Transgenic Mice Expressing Human Angiotensin-Converting Enzyme 2. Cell.

[43] Lian H (2018). The Zinc-Finger Protein ZCCHC3 Binds RNA and Facilitates Viral RNA Sensing and Activation of the RIG-I-like Receptors. Immunity.

[44] Fu YZ (2017). Human Cytomegalovirus Tegument Protein UL82 Inhibits STING-Mediated Signaling to Evade Antiviral Immunity. Cell Host Microbe.

